# Evaluation of health-related quality of life in children with postoperative delirium after surgical repair of ventricular septal defect: short- and mid-term follow-up

**DOI:** 10.1186/s12887-023-03843-3

**Published:** 2023-02-11

**Authors:** Jiang-Shan Huang, Wen-Hao Lin, Yu-Kun Chen

**Affiliations:** Department of Cardiac Surgery, College of Clinical Medicine for Obstetrics & Gynecology and Pediatrics, Fujian Children’s Hospital (Fujian Branch of Shanghai Children’s Medical Center), Fujian Medical University, Fuzhou, China

## Abstract

**Background:**

To investigate health-related quality of life (HRQOL) in children with postoperative delirium (POD) after surgical repair of ventricular septal defects (VSDs).

**Methods:**

A total of 109 patients were enrolled and assigned to the POD group (*n* = 47) and the non-POD group (*n* = 62). HRQOL was assessed by the PedsQLTM 3.0 Cardiac Module at discharge, and at the three- and six-month postoperative follow.

**Results:**

Significant differences were detected in age, operation time, CPB time, mechanical ventilation duration, and length of ICU stay between the two groups, whereas sex and the VSD size did not significantly differ between groups. In terms of “cardiac heart problems and treatment” and “treatment-II”, the HRQOL scores of the non-POD group were significantly better than those of the POD group. In terms of “perceived physical appearance” and “treatment anxiety”, the POD group had significantly higher scores than the non-POD group at discharge and at the three-month postoperative follow-up. In terms of “cognitive problems” and “communication”, the occurrence of POD still affected HRQOL at three months postoperatively, but the effect was significantly reduced at six months postoperatively. In terms of “total scores”, both groups scored increasingly higher over time. The non-POD group had higher scores at discharge and three months postoperatively than the POD group, but no significant difference persisted at six months postoperatively.

**Conclusion:**

During the follow-up period, the HRQOL of the children with POD after surgical repair of VSD was inferior to that of the children without POD at discharge and three months postoperatively. However, the HRQOL did not differ between the two groups at six months postoperatively.

## Introduction

Delirium is a common central nervous system dysfunction after cardiac surgery [1]. A recent study showed a prevalence of 49% in pediatric cardiac intensive care units. Adult delirium is associated with increased mortality, longer hospital stays, postdischarge morbidity, decreased neurocognitive function, and decreased health-related quality of life (HRQOL) [2, 3]. Critically ill children often need to be treated with sedative drugs and are at risk for hallucinations and altered thinking [4]. Thus, experiences of postoperative delirium (POD) after cardiac surgery might prevent children and their families from returning to normal activities and expose them to constant worry and changing expectations about the future [5]. Few studies have focused on HRQOL in children with POD after surgical repair of ventricular septal defects (VSDs). Therefore, this study aimed to investigate HRQOL in children with POD after VSD, surgical repair to provide a reference for the early recovery of children with POD and create a good growth environment.

## Patients and methods

### Patients

This study was retrospective. We enrolled 137 children who underwent surgical VSD repair between January 2020 and February 2022. The inclusion criteria were patients with simple VSD who received surgical repair, without other operations. Exclusion criteria: (1) other heart malformations (*n* = 12); (2) preoperative complications, such as severe pulmonary infection or other organ failures (*n* = 4); (3) severe postoperative complications (conduction block; secondary thoracotomy, etc.) (*n* = 1); (4) significant postoperative residual shunt (*n* = 2); (5) combined chromosomal and other physical abnormalities (*n* = 3); (6) brain injury (traumatic brain injury, cerebral hemorrhage, brain surgery, etc.) (*n* = 1); (7) patients with incomplete data or lost to follow-up (*n* = 5).

### Measurement of delirium

The Cornell Assessment of Pediatric Delirium scale (CAPD) is a reliable and simple bedside tool for children of all ages, and a score greater than 9 is considered to indicate delirium [6]. Each item of the CAPD score scale is scored 0–4 points according to the frequency of the child’s behaviors using the Likert 5-level scoring method. Sedative drugs are often used after heart surgery. Therefore, combined with the Richmond Agitation-Sedation Scale (RASS), the CAPD can improve the ability to assess delirium in severely ill children. The RASS is a reliable scale for assessing the quality of sedation and is suitable for critically ill patients [7]. Since the design of CAPD considers an extended observation period (rather than as a point-of-time screening), the assessment time was at the end of the nurse’s shift.

### Measurement of HRQOL

The Chinese version of the PedsQL™ 3.0 Cardiac Module was used to evaluate HRQOL in 2- to 4-year-old children, with postoperative delirium after surgical VSD repair. The PedsQL™ 3.0 Cardiac Module is a commonly used scale for measuring the quality of life of children with heart disease. After repeated evaluation and application, it has been proven to have good reliability and validity [8]. The scale is divided into six different dimensions, each of which contains different items. Each item asks about the frequency of an event in the recent month, and its answer options are divided into five grades of “0 ~ 4”, which are translated into 100 ~ 0 points in statistical scoring: “0” (100 points) means “never”, “1” (75 points) means “hardly”, “2” (50 points) means “sometimes”, “3” (25 points) means “often”, and “4” (0 points) means “always”. The score for each dimension is the sum of the scores of the items under that dimension divided by the number of items contained. The total score of the scale is the sum of the scores of each item divided by the total number of items on the scale. The total score and the score of each dimension ranged from 0 to 100, and a higher score indicates a better quality of life [8].

At our center, the children were informed of the necessity and time of postoperative review when they were discharged from the hospital. Questionnaires were administered at discharge, three months postoperatively, and six months postoperatively. Specialized follow-up medical staff would cooperate with the children and their families to complete the questionnaire on quality of life, and other specialized staff statistically analyzed the data. The patients and their families had good compliance.

### Statistical method

SPSS Statistics 23 was used for statistical analysis. The normality of count data in the basic data was tested and showed that data were normally distributed; consequently a t test was adopted. Measured data were analyzed with the chi-square test. Analysis of variance was used to compare the HRQOL between the two groups, and LSD was used for the post hoc test.

## Result

Based on the inclusion and exclusion criteria, 109 patients were enrolled and assigned to the POD group (*n* = 47) and the no-POD group (*n* = 62) based on the evaluation of delirium. In terms of clinical data, age, operation time, CPB time, mechanical ventilation duration, and the length of ICU stay significantly differed between the two groups, whereas gender and the VSD size did not significantly differ between groups (Table 1).

**Table 1:** General clinical data of patients

Item	POD	No-POD	*p*
Number	47	62	/
M/F	22/25	34/28	0.524
Age (m)	31.8 ± 10.6	36.5 ± 12.9	0.022
Size of VSD (mm)	5.5 ± 1.7	5.1 ± 1.5	0.128
CPB (min)	65.3 ± 10.8	60.7 ± 12.5	0.023
Operation time (min)	133.6 ± 38.5	118.0 ± 30.0	0.008
Mechanical ventilation	14.5 ± 4.3	11.8 ± 4.4	0.000
ICU(d)	3.4 ± 1.4	2.5 ± 1.7	0.017

Detailed data on HRQOL are shown in Table 2. (1) In terms of “total scores”, the two groups scored increasingly higher over time. The non-POD group had higher scores than the POD group at discharge and three months postoperatively, but this difference did not persist at six months postoperatively (*P* = 0.061). (2) In terms of “heart problems and treatment”, the two groups both showed an upward trend, and a significant difference was detected between groups. The quality of life was higher in the non-POD group than in the POD group at discharge and at three months and six months postoperatively, and the difference was statistically significant. (3) In terms of “treatment II”, the intragroup comparison showed a significant difference between discharge and three months postoperatively. A significant difference was detected between the two groups at discharge, but this difference did not persist at three months postoperatively (*P* = 0.20). (4) In terms of “perceived physical appearance”, both groups showed an upward trend, but no significant difference was detected between postoperative three months and postoperative six months (POD: *P* = 0.18; no-POD: *P* = 0.16). Intergroup comparisons showed a higher quality of life in the no-POD group than in the POD group at discharge, but this difference did not persist at three months (*P* = 0.25) six months postoperatively (*P* = 0.47). (5) In terms of “treatment anxiety”, the two groups also showed an upward trend, and a significant difference was detected. A comparison between the two groups showed that, the POD group had lower scores than the non-POD at discharge and three months postoperatively. At six months postoperatively, no significant difference was detected between the two groups (*P* = 0.198). (6) In terms of “cognitive problems”, both groups also showed an upward trend, but no significant difference was detected between postoperative three months and postoperative six months (POD: *P* = 0.184; non-POD: *P* = 0.515). A comparison between the two groups showed that, the POD group had lower scores at discharge and three months postoperatively, but this difference did not persist at six months postoperatively (*P* = 0.104). (7) In terms of “communication”, the two groups scored increasingly higher over time. The non-POD group had higher scores than the POD group at discharge and three months postoperatively, but no significant difference was detected at six months postoperatively (*P* = 0.61).

**Table 2:** Comparison of score of the PedsQLTM 3.0 Cardiac Module

Item	POD			No-POD		
	Discharged	POM3	POM6	Discharged	POM3	POM6
Total score	51 ± 14	62.8 ± 13.7	72.0 ± 15.2^b^	56.6 ± 13.9	67.2 ± 10.3	74.3 ± 10.0^b^
Heart problems and treatment	61.2 ± 12.8	70.4 ± 10.2	78.4 ± 17.6	66.8 ± 17.3	73.4 ± 15.9	84.5 ± 19.0
Treatment II	47.7 ± 12.6	64.7 ± 12.4^b^	/	52.4 ± 11.7	67.2 ± 11.1^b^	/
Perceived physical appearance	51.0 ± 14.3	68.7 ± 12.6^a^^b^	71.9 ± 13.6^ab^	55.6 ± 12.6	71.0 ± 10.8^ab^	73.4 ± 11.2^ab^
Treatment anxiety	47.2 ± 14.6	53.6 ± 14.2	69.3 ± 16.9^b^	53.7 ± 16.5	60.9 ± 15.4	72.9 ± 15.1^b^
Cognitive problems	52.3 ± 11.5	63.3 ± 10.6^a^	66.2 ± 13.3^ab^	57.2 ± 12.7	69.1 ± 10.3^a^	70.6 ± 17.5^ab^
Communication	50.1 ± 13.6	56 ± 12.6	69.3 ± 10.8^b^	54 ± 13.9	61.7 ± 10.3	70.2 ± 9.9^b^

POM3: postoperative three months

POM6: postoperative six months

^a^Indicates that there was no significant statistical difference in post-test within the group

^b^Indicates that there is no significant statistical difference between the two groups at the same time

## Discussion

Childhood delirium is a state of acute brain dysfunction common in pediatric intensive care units [9]. The occurrence of delirium is not conducive to the recovery of the autonomic nervous system and the primary disease and may lead to adverse events such as unplanned extubation, prolonged hospital stays, and excessive medical expenses [10–12]. Elderly individuals, infants, and children are more susceptible to delirium. Children with delirium usually have a reduced ability to maintain and transfer attention, weakened orientation to the surrounding environment, memory, language disorders, hallucinations, and other clinical manifestations [13]. Critically ill children often undergo multiple treatments, are treated with sedative drugs, and are at risk for hallucinations and altered thinking [4]. Children may also have subtle and persistent sensory-motor and behavioral disturbances after acute delirium subsides [5]. As Chinese society has become more developed, Chinese have given increasing attention to the postoperative quality of life of children. However, few studies have focused on the quality of life of children with delirium after surgical VSD repair. Therefore, this study aimed to investigate the quality of life of children with delirium after surgical VSD repair.

In recent years, statistical data on the ages of children with VSD treated at in our center showed that, most of the patients were in the 2–4-year-old group, followed by the 5–7-year-old group, and VSD was very rare in the 8–12-year-old group. These data did not include severe VSD in children aged up to 1 year old. In the selection of age groups in this study, we fully considered the age composition characteristics of our center, and the requirements of PedsQL™ 3.0 Cardiac Module on age. In addition, the personalities of children aged 2–4 also rapidly develop. Therefore, we selected children aged 2–4 as our research objects. Delirium was assessed using the CAPD scale, a reliable and simple bedside tool for children of all ages [6]. In 2016, the CAPD was recommended by the European Society of Pediatric and Neonatal Intensive Care as an assessment tool for delirium in children and this tool has an A evidence level [14, 15]. Sedative drugs are often used after heart surgery. Therefore, combined with the Richmond Agitation-Sedation Scale, the CAPD can improve the ability to assess delirium in severely ill children.

In recent years, increasingly attention has been given to research on children’s quality of life [16]. In this paper, the PedsQLTM 3.0 Cardiac Module, which is a commonly used measure of the quality of life for children with heart disease, was selected to evaluate the HRQOL. After repeated evaluation and application, it was proven to have good reliability and validity [17]. The content of the scale can be used to comprehensively evaluate quality of life from three main aspects: physical, psychological, and social functions. (1) In the first scale, two dimensions of “cardiac heart problems and treatment” and “treatment-II” are used to evaluate physiological function, and these dimensions constitute the basis that affects quality of life. In this respect, the HRQOL of the two groups improved gradually over time, and the HRQOL of the no-POD group was significantly better than that of the POD group. We hypothesized that although the anatomic malformations had been corrected by surgery, the mental stress of the patients in the POD group was more obvious, leading the children and their parents to give excessive attention to cardiac symptoms, thus affecting their quality of life. (2) In the second scale, “perceived physical appearance” and “treatment anxiety” reflect the psychological function of the children. To some extent, these two aspects reflect the differences in the psychological function of children before and after treatment. This study showed that the children had more medical fear after surgery, and the patients in the POD group had significantly higher medical fear than those in the no-POD group three months after surgery. With the increase in age at the six-month follow up, no significant difference was detected between the two groups. Children experience the entire process of hospitalization, infusion, surgery, and monitoring, which undoubtedly cause great psychological trauma to children. Therefore, future work should focus on how to reduce early postoperative adverse psychological stimulation for children, create a warmer and more comfortable medical environment, strengthen psychological counseling for children with perioperative cardiac surgery, and strengthen psychological counseling for parents. These measures are of great significance for our clinical work. (3) In the third scale, two dimensions of “cognitive problems” and “communication” are used to evaluate social functions. In terms of social function, the occurrence of POD still affected the postoperative quality of life during the three-month follow-up, but the effect was significantly reduced during the three-month follow-up. POD group was more likely to affect children’s social-cognitive function in the early postoperative period. Although the social function of children also depended on the family and social education received after surgery, parents could give good and correct guidance to children during treatment, which had an important role in the recovery and improvement of children’s social cognitive function. (4) In terms of overall quality of life, postoperative delirium in children gradually reduced the psychological and physiological effects of time, postoperative quality of life in children significantly improved, and the postoperative quality of life did not significantly differ between the groups during the six-month follow-up. However, whether it had an impact on the long-term quality of life still needs further research.

This work was a retrospective analysis conducted at a single center with a small sample. Family members had a high follow-up rate and a good degree of cooperation, and the questionnaire was collected by a specialized team. Therefore, the data were reliable. However, the follow-up period of this study was short, and the follow-up period should consequently be increased in the future to explore the influence of postoperative delirium on long-term quality of life. In this study, subjects were aged from 2 to 4 years old, constituting a relatively narrow range. In the future, the age range should be expanded for further exploration.

## Conclusion

The HRQOL of children after surgical VSD repair improved over time. The HRQOL of children with POD was inferior to that of the children without POD at discharge and the three-month postoperative follow-up. However, HRQOL did not differ between the two groups at the six-month postoperative follow-up. 

## Data Availability

The datasets used and analyzed during the current study are available from the corresponding author on reasonable request.
